# Supplementary material for: Fluorescence vs. Phosphorescence: Which Scenario Is Preferable in Au(I) Complexes with Benzothiadiazoles?
Source: Molecules. 2022 Nov 23;27(23):8162. doi: 10.3390/molecules27238162 (PMC9741114; doi:10.3390/molecules27238162)
Supplement: Supplementary file 1 [file molecules-27-08162-s001.zip › molecules-2049469-supplementary.pdf]

Supporting Information

Fluorescence vs Phosphorescence: which scenario is preferable in Au(I) complexes with benzothiadiazoles?

R.M. Khisamov, A.A. Ryadun, S.N. Konchenko, T.S. Sukhikh*

Figure S1. Simulated and experimental powder XRD patterns for compound **1** (Cu K_α radiation). 2

Figure S2. Simulated and experimental powder XRD patterns for compound **2a** (Cu K_α radiation). 2

Figure S3. Simulated and experimental powder XRD patterns for compound **2b** (Cu K_α radiation). 2

Table S1 – Vertical S_0–S_n (n = 1–10) transitions for **1** in the ground state 3

Table S2 – Vertical S_0–T_n (n = 1–10) transitions for **1** in the ground state 3

Table S3 – Vertical S_0–S_n (n = 1–10) transitions for **2a** in the ground state 6

Table S4 – Vertical S_0–T_n (n = 1–10) transitions for **2a** in the ground state 7

Table S5 – Vertical S_0–S_n (n = 1–10) transitions for **2b** in the ground state 9

Table S6 – Vertical S_0–T_n (n = 1–10) transitions for **2b** in the ground state 10

Figure S4. Frontier orbitals of S_0 equilibrium geometry of **1** 12

Figure S5. Frontier orbitals of S_0 equilibrium geometry of **2a** and **2b** 13

Figure S6. UV-Vis, emission and excitation spectra of solids **1**, **2a** and **2b**. 13

Figure S7. UV-Vis, emission and excitation spectra of **2a** and **2b** in THF 14

Figure S8. Emission decay kinetics and single exponential fit for compound **1**..14

Figure S9. Emission decay kinetics and single exponential fit for compound **2a**......................................15

Figure S10. Emission decay kinetics and double exponential fit for compound **2b** 15

Figure S11. Decreasing intensity of the emission bands for compounds **1** and **2a** in the temperature range 77–300 K 16

Table S7. Values of selected spin-orbit coupling matrix elements for the complexes. 16

Table S8. Values of selected spin-orbit coupling matrix elements for complex **1** at excited states geometries 16

Figure S12. Comparison of LUMO and HOMO orbitals of **1** in S_0, S_1, T_1, and T_2 equilibrium geometries...17

Figure S13. Representation of energy levels for different geometry states (S_o, S_1, T_1 and T_2) of complexes **1**–**2** in the energy range from 0 to $30\cdot10^3$ cm^{-1}..17

Figure S14. IR-spectra of compounds **1**–**2** recorded in KBr pellets..18

Figure S15. Comparison of equilibrium geometries of complexes **1**, **2a** and **2b** in different states...........18

Figure S16. Overlaid geometries for S_1 equilibrium state of **2b** obtained by two different DFT methods.18

Table S9. Crystal data and structure refinement for the compounds. 19

Table S10. Selected bond lengths for structures **1**–**2**. 19

Table S11. Selected bond angles for structures **1**–**2**. 20

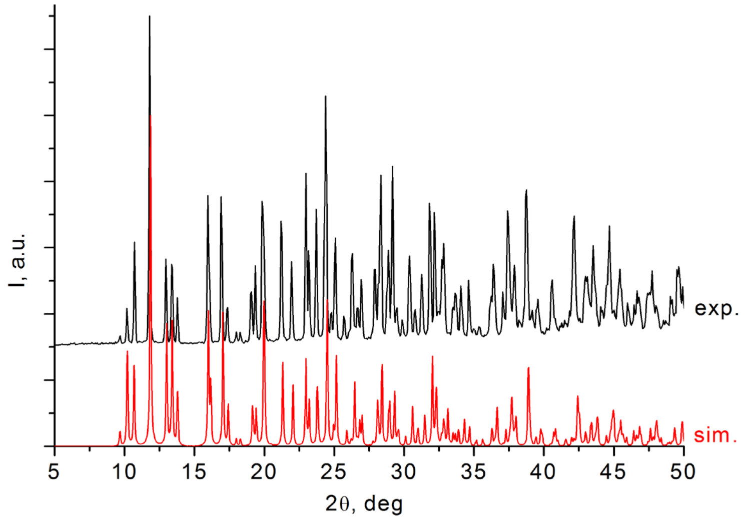

Figure S1. Simulated and experimental powder XRD patterns for compound **1** (Cu K_{α} radiation).

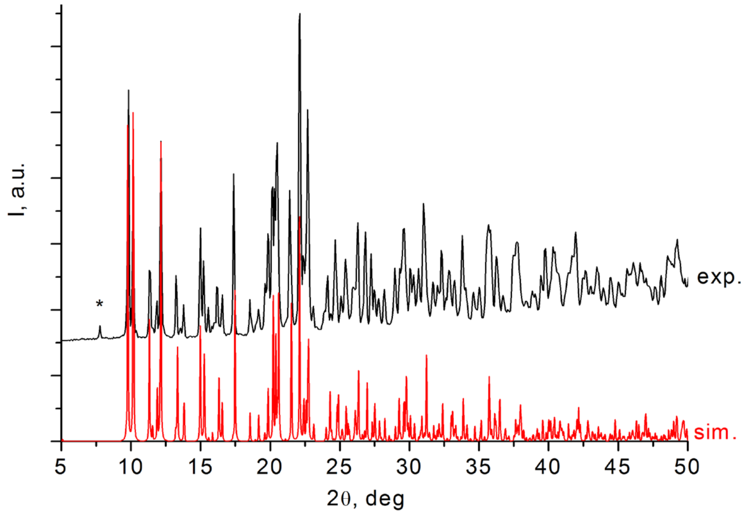

Figure S2. Simulated and experimental powder XRD patterns for compound **2a** (Cu K_{α} radiation). Peak attributed to a very minor quantity of **2b** is highlighted by "*".

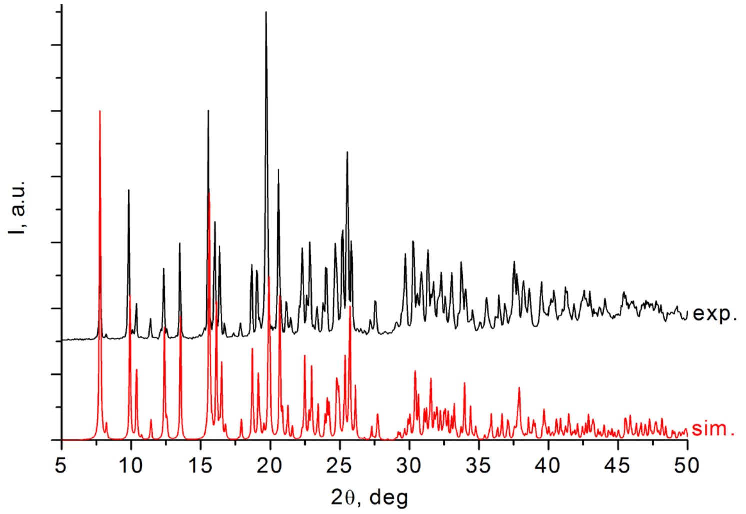

Figure S3. Simulated and experimental powder XRD patterns for compound **2b** (Cu K_{α} radiation).

Table S1 – Vertical S_0–S_n (n = 1–10) transitions for **1** in the ground state (TD-PBE0-D3BJ/def2-TZVP(-f))

State	Energy, cm-1	Wavelength, nm	Oscilator strength	Transitions	Contributions
1	25991.4	384.7	0.094493292	H → L	0.982100
2	31902.4	313.5	0.007782581	H → L + 1	0.975042
3	32655.4	306.2	0.004235967	H - 1 → L	0.987044
4	32879.1	304.1	0.004122596	H - 2 → L	0.989941
5	35359.3	282.8	0.023497613	H - 3 → L<br>H → L + 2	0.032822<br>0.930323
6	35949.5	278.2	0.042800164	H - 3 → L<br>H → L + 2<br>H → L + 5	0.912618<br>0.034410<br>0.014070
7	37164.6	269.1	0.003285502	H - 2 → L + 1<br>H - 1 → L + 1	0.163619<br>0.799327
8	37326.2	267.9	0.087819734	H - 4 → L<br>H → L + 4<br>H → L + 5<br>H → L + 7<br>H → L + 9	0.786338<br>0.033176<br>0.086942<br>0.033368<br>0.010734
9	37682.3	265.4	0.015908996	H - 2 → L + 1<br>H - 1 → L + 1	0.800502<br>0.163600
10	38244.8	261.5	0.053996018	H → L + 3<br>H → L + 4	0.911014<br>0.062411

Table S2 – Vertical S_0–T_n (n = 1–10) transitions for **1** in the ground state (TDA-PBE0-D3BJ/def2-TZVP(-f))

State	Energy, cm-1	Wavelength, nm	Transitions	Contributions
1	18684.8	535.2	H - 9 → L<br>H → L	0.010633 (c= 0.10311461)<br>0.917438 (c= 0.95782991)
2	26994.8	370.4	H - 6 → L<br>H - 4 → L<br>H - 3 → L<br>H - 1 → L	0.016917 (c= -0.13006692)<br>0.705779 (c= -0.84010642)<br>0.211080 (c= -0.45943457)<br>0.013973 (c= 0.11820715)
3	30944.4	323.2	H - 13 → L<br>H - 9 → L<br>H - 5 → L + 1<br>H - 3 → L<br>H - 3 → L + 1<br>H → L + 1<br>H → L + 5	0.014882 (c= -0.12199138)<br>0.010935 (c= 0.10456840)<br>0.015091 (c= -0.12284598)<br>0.014558 (c= -0.12065808)<br>0.033013 (c= 0.18169347)<br>0.784469 (c= 0.88570247)<br>0.015244 (c= 0.12346626)

4	31902.4	313.5	H - 9 → L + 1<br>H - 9 → L + 2<br>H - 8 → L + 1<br>H - 8 → L + 2<br>H - 7 → L + 4<br>H - 6 → L + 3<br>H - 6 → L + 4<br>H - 5 → L + 1<br>H - 5 → L + 2<br>H - 3 → L + 1<br>H - 3 → L + 2<br>H - 2 → L<br>H - 1 → L + 1<br>H → L + 1	0.028168 (c= -0.16783316)<br>0.067330 (c= -0.25948051)<br>0.015903 (c= 0.12610895)<br>0.023720 (c= -0.15401222)<br>0.026628 (c= -0.16318200)<br>0.089239 (c= -0.29872874)<br>0.062079 (c= 0.24915726)<br>0.148262 (c= 0.38504790)<br>0.050872 (c= 0.22554766)<br>0.148932 (c= -0.38591671)<br>0.057452 (c= -0.23969089)<br>0.023477 (c= 0.15322317)<br>0.010649 (c= 0.10319436)<br>0.063729 (c= 0.25244597)
5	32341.2	309.2	H - 13 → L<br>H - 9 → L + 1<br>H - 8 → L<br>H - 8 → L + 1<br>H - 8 → L + 2<br>H - 8 → L + 4<br>H - 7 → L + 2<br>H - 7 → L + 3<br>H - 7 → L + 4<br>H - 6 → L + 4<br>H - 5 → L + 2<br>H - 4 → L<br>H - 3 → L + 2<br>H - 2 → L<br>H - 1 → L<br>H - 1 → L + 1	0.017358 (c= -0.13174937)<br>0.035493 (c= 0.18839549)<br>0.015806 (c= -0.12572003)<br>0.124105 (c= 0.35228566)<br>0.024688 (c= -0.15712301)<br>0.017115 (c= -0.13082471)<br>0.010778 (c= 0.10381543)<br>0.066859 (c= -0.25857013)<br>0.022506 (c= -0.15002140)<br>0.029045 (c= -0.17042566)<br>0.057844 (c= -0.24050724)<br>0.015258 (c= 0.12352259)<br>0.041104 (c= 0.20274094)<br>0.197334 (c= 0.44422348)<br>0.163197 (c= 0.40397604)<br>0.010211 (c= -0.10104796)
6	32535.5	307.4	H - 13 → L<br>H - 9 → L<br>H - 9 → L + 1<br>H - 8 → L + 1<br>H - 8 → L + 2<br>H - 8 → L + 4<br>H - 7 → L + 2<br>H - 7 → L + 3<br>H - 7 → L + 4<br>H - 6 → L + 4<br>H - 5 → L + 2<br>H - 4 → L<br>H - 3 → L<br>H - 3 → L + 2<br>H - 2 → L<br>H - 1 → L<br>H → L + 1	0.015911 (c= 0.12613874)<br>0.017875 (c= -0.13369790)<br>0.014071 (c= 0.11862268)<br>0.129455 (c= 0.35979892)<br>0.025722 (c= -0.16038008)<br>0.014265 (c= -0.11943805)<br>0.015288 (c= 0.12364347)<br>0.061245 (c= -0.24747765)<br>0.024738 (c= -0.15728404)<br>0.014733 (c= -0.12138015)<br>0.037869 (c= -0.19460063)<br>0.031085 (c= -0.17630993)<br>0.012701 (c= 0.11270063)<br>0.024800 (c= 0.15747900)<br>0.192463 (c= -0.43870571)<br>0.202593 (c= -0.45010324)<br>0.047320 (c= 0.21753263)

7	32687.5	305.9	H - 2 → L<br>H - 1 → L	0.395822 (c= -0.62914387)<br>0.558561 (c= 0.74736944)
8	33545.4	298.1	H - 17 → L<br>H - 16 → L<br>H - 13 → L<br>H - 12 → L<br>H - 9 → L<br>H - 8 → L<br>H - 7 → L<br>H - 5 → L<br>H - 4 → L<br>H - 3 → L<br>H - 2 → L<br>H - 1 → L<br>H → L + 1<br>H → L + 2<br>H → L + 5<br>H → L + 6<br>H → L + 8<br>H → L + 9	0.024004 (c= 0.15493069)<br>0.015549 (c= 0.12469607)<br>0.154157 (c= -0.39262803)<br>0.015975 (c= 0.12639410)<br>0.091876 (c= 0.30311043)<br>0.031985 (c= -0.17884238)<br>0.012006 (c= 0.10957331)<br>0.011758 (c= 0.10843530)<br>0.026293 (c= 0.16215238)<br>0.125419 (c= -0.35414516)<br>0.146619 (c= -0.38290803)<br>0.039709 (c= -0.19927069)<br>0.040428 (c= -0.20106771)<br>0.070254 (c= -0.26505393)<br>0.012077 (c= 0.10989693)<br>0.014386 (c= -0.11994237)<br>0.026263 (c= -0.16205773)<br>0.053090 (c= 0.23041308)
9	34602.6	289.0	H - 12 → L<br>H - 12 → L + 8<br>H - 12 → L + 9	0.905087 (c= -0.95136042)<br>0.010232 (c= 0.10115372)<br>0.014920 (c= -0.12214720)
10	34910.1	286.4	H - 13 → L<br>H - 9 → L<br>H - 7 → L + 1<br>H - 7 → L + 2<br>H - 6 → L + 1<br>H - 4 → L<br>H - 3 → L<br>H → L + 2	0.012819 (c= -0.11322052)<br>0.012287 (c= 0.11084876)<br>0.010890 (c= 0.10435563)<br>0.015327 (c= -0.12380068)<br>0.030725 (c= 0.17528632)<br>0.017822 (c= 0.13350056)<br>0.038770 (c= -0.19690141)<br>0.777019 (c= 0.88148662)

Table S3 – Vertical S_0–S_n (n = 1–10) transitions for **2a** in the ground state (TD-PBE0-D3BJ/def2-TZVP(-f))

State	Energy, cm-1	Wavelength, nm	Oscilator strength	Transitions	Contributions
1	25077.9	398.8	0.099372434	H → L	0.982747
2	31090.0	321.6	0.001508312	H → L + 1<br>H → L + 2	0.969543<br>0.015382
3	33368.4	299.7	0.015002746	H → L + 1<br>H → L + 2	0.015390<br>0.971676
4	35135.9	284.6	0.001394731	H - 2 → L<br>H - 1 → L	0.421173<br>0.568399
5	35187.4	284.2	0.000200988	H - 2 → L<br>H - 1 → L	0.567817<br>0.427491
6	35454.4	282.1	0.004228600	H → L + 3<br>H → L + 4	0.753663<br>0.227395
7	35875.6	278.7	0.012174478	H - 5 → L<br>H - 4 → L<br>H - 3 → L<br>H → L + 3<br>H → L + 4<br>H → L + 5<br>H → L + 6	0.024197<br>0.018897<br>0.036375<br>0.192879<br>0.633515<br>0.024147<br>0.038656
8	36877.1	271.2	0.034964954	H - 5 → L<br>H - 4 → L<br>H - 3 → L<br>H → L + 4<br>H → L + 5<br>H → L + 6<br>H → L + 8	0.015452<br>0.021815<br>0.753294<br>0.069321<br>0.019278<br>0.040181<br>0.036999
9	37140.2	269.2	0.013706441	H - 3 → L<br>H → L + 5<br>H → L + 6	0.089488<br>0.207455<br>0.681612
10	37616.3	265.8	0.057442744	H - 6 → L<br>H - 5 → L<br>H - 4 → L<br>H - 3 → L<br>H → L + 4<br>H → L + 5<br>H → L + 6<br>H → L + 7<br>H → L + 8<br>H → L + 9	0.015618<br>0.257313<br>0.428207<br>0.085696<br>0.023838<br>0.018617<br>0.014811<br>0.017556<br>0.092361<br>0.011423

Table S4 – Vertical S_0–T_n (n = 1–10) transitions for **2a** in the ground state (TDA-PBE0-D3BJ/def2-TZVP(-f))

State	Energy, cm-1	Wavelength, nm	Transitions	Contributions
1	18056.1	553.8	H - 11 → L<br>H → L<br>H → L + 11	0.015391 (c= 0.12405968)<br>0.934472 (c= -0.96668102)<br>0.013682 (c= -0.11696919)
2	27506.2	363.6	H - 11 → L<br>H - 6 → L<br>H - 5 → L<br>H - 4 → L<br>H - 3 → L	0.010648 (c= 0.10319143)<br>0.027223 (c= -0.16499529)<br>0.510131 (c= -0.71423442)<br>0.272330 (c= -0.52185250)<br>0.142976 (c= -0.37812173)
3	30530.6	327.5	H - 10 → L + 2<br>H - 9 → L + 5<br>H - 8 → L + 3<br>H - 7 → L + 1<br>H - 6 → L + 1<br>H - 4 → L + 1<br>H - 3 → L + 1<br>H → L + 1<br>H → L + 2	0.031353 (c= 0.17706665)<br>0.010447 (c= -0.10221107)<br>0.017067 (c= 0.13064006)<br>0.016472 (c= -0.12834204)<br>0.036620 (c= 0.19136278)<br>0.013702 (c= -0.11705633)<br>0.011838 (c= 0.10880166)<br>0.762307 (c= -0.87310173)<br>0.025665 (c= 0.16020175)
4	31905.8	313.4	H - 12 → L + 2<br>H - 11 → L<br>H - 10 → L + 1<br>H - 9 → L + 3<br>H - 8 → L + 5<br>H - 7 → L + 2<br>H - 7 → L + 3<br>H - 6 → L + 1<br>H - 6 → L + 2<br>H - 4 → L + 2<br>H - 3 → L + 2<br>H → L + 1<br>H → L + 2	0.014133 (c= 0.11888309)<br>0.010474 (c= -0.10234444)<br>0.173398 (c= -0.41641111)<br>0.080672 (c= -0.28402785)<br>0.060440 (c= 0.24584521)<br>0.050875 (c= 0.22555408)<br>0.010189 (c= -0.10094094)<br>0.020409 (c= -0.14285936)<br>0.122619 (c= -0.35016980)<br>0.033604 (c= 0.18331512)<br>0.025935 (c= -0.16104218)<br>0.018216 (c= 0.13496780)<br>0.225290 (c= 0.47464747)
5	32389.2	308.7	H - 12 → L + 1<br>H - 10 → L + 2<br>H - 9 → L + 5<br>H - 8 → L + 3<br>H - 7 → L + 1<br>H - 7 → L + 3<br>H - 6 → L + 1<br>H - 6 → L + 5<br>H - 4 → L + 1<br>H - 3 → L + 1<br>H - 1 → L + 1<br>H → L + 1<br>H → L + 4	0.018683 (c= -0.13668472)<br>0.123176 (c= 0.35096458)<br>0.055566 (c= -0.23572336)<br>0.083039 (c= 0.28816424)<br>0.049148 (c= -0.22169328)<br>0.012285 (c= 0.11083752)<br>0.156421 (c= 0.39550075)<br>0.018161 (c= -0.13476271)<br>0.028604 (c= -0.16912826)<br>0.049921 (c= 0.22343100)<br>0.014286 (c= 0.11952299)<br>0.177467 (c= 0.42126835)<br>0.015857 (c= 0.12592568)

6	32488.6	307.8	H - 7 → L + 4<br>H - 6 → L + 1<br>H - 6 → L + 4<br>H - 6 → L + 6<br>H - 6 → L + 9<br>H - 5 → L + 4<br>H - 5 → L + 6<br>H - 4 → L + 6<br>H - 3 → L + 4<br>H - 3 → L + 7<br>H - 2 → L + 4<br>H - 1 → L + 6	0.011356 (c= -0.10656677)<br>0.011961 (c= -0.10936839)<br>0.061854 (c= -0.24870517)<br>0.031157 (c= 0.17651397)<br>0.013505 (c= -0.11621005)<br>0.026799 (c= -0.16370466)<br>0.105783 (c= -0.32524361)<br>0.129971 (c= 0.36051554)<br>0.334072 (c= 0.57798996)<br>0.017990 (c= -0.13412793)<br>0.017270 (c= 0.13141598)<br>0.010040 (c= 0.10020109)
7	32763.2	305.2	H - 19 → L<br>H - 18 → L<br>H - 12 → L<br>H - 11 → L<br>H - 10 → L<br>H - 10 → L + 1<br>H - 9 → L<br>H - 8 → L<br>H - 8 → L + 5<br>H - 7 → L<br>H - 6 → L + 2<br>H - 5 → L<br>H - 3 → L<br>H → L<br>H → L + 2<br>H → L + 4<br>H → L + 8<br>H → L + 9<br>H → L + 11	0.021590 (c= 0.14693459)<br>0.041844 (c= -0.20455912)<br>0.021070 (c= 0.14515375)<br>0.361799 (c= -0.60149749)<br>0.030625 (c= 0.17500063)<br>0.017997 (c= 0.13415296)<br>0.011014 (c= -0.10494885)<br>0.018185 (c= 0.13485172)<br>0.013995 (c= -0.11830186)<br>0.010249 (c= -0.10123929)<br>0.016001 (c= 0.12649518)<br>0.010282 (c= -0.10140093)<br>0.029372 (c= 0.17138313)<br>0.010413 (c= -0.10204169)<br>0.054508 (c= 0.23346851)<br>0.018666 (c= 0.13662257)<br>0.028429 (c= 0.16860841)<br>0.016437 (c= -0.12820733)<br>0.110105 (c= -0.33182146)
8	33489.4	298.6	H - 11 → L<br>H - 10 → L + 1<br>H - 9 → L + 3<br>H - 8 → L + 5<br>H - 6 → L + 2<br>H → L + 1<br>H → L + 2<br>H → L + 4<br>H → L + 6<br>H → L + 8	0.054213 (c= -0.23283659)<br>0.030704 (c= -0.17522680)<br>0.028686 (c= -0.16937026)<br>0.026727 (c= 0.16348335)<br>0.022794 (c= -0.15097570)<br>0.012251 (c= -0.11068385)<br>0.637312 (c= -0.79831816)<br>0.027159 (c= 0.16480078)<br>0.011362 (c= 0.10659326)<br>0.025250 (c= 0.15890254)
9	34400.2	290.7	H - 19 → L<br>H - 18 → L<br>H - 11 → L<br>H → L + 2<br>H → L + 3<br>H → L + 4<br>H → L + 6	0.029131 (c= -0.17067738)<br>0.027403 (c= 0.16553911)<br>0.018433 (c= 0.13576823)<br>0.026337 (c= 0.16228638)<br>0.010930 (c= -0.10454669)<br>0.159880 (c= 0.39984946)<br>0.084629 (c= 0.29091027)

			H → L + 7<br>H → L + 8<br>H → L + 9<br>H → L + 11<br>H → L + 12	0.087418 (c= -0.29566532)<br>0.400590 (c= 0.63292142)<br>0.017085 (c= -0.13071074)<br>0.038992 (c= 0.19746361)<br>0.012312 (c= -0.11095741)
10	34586.7	289.1	H - 13 → L<br>H - 13 → L + 11<br>H - 12 → L	0.751403 (c= -0.86683514)<br>0.022772 (c= 0.15090510)<br>0.174561 (c= -0.41780491)

Table S5 – Vertical S_0–S_n (n = 1–10) transitions for **2b** in the ground state (TD-PBE0-D3BJ/def2-TZVP(-f))

State	Energy, cm-1	Wavelength, nm	Oscilator strength	Transitions	Contributions
1	24367.9	410.4	0.093296026	H → L	0.983738
2	32361.6	309.0	0.013422341	H → L + 1	0.987182
3	33674.8	297.0	0.005042399	H → L + 2	0.983923
4	33998.1	294.1	0.000174909	H - 3 → L<br>H - 1 → L	0.020807<br>0.971867
5	34118.5	293.1	0.000143744	H - 4 → L<br>H - 3 → L<br>H - 2 → L	0.016160<br>0.038600<br>0.939219
6	34810.7	287.3	0.004917565	H - 4 → L<br>H - 3 → L<br>H - 2 → L<br>H - 1 → L	0.020252<br>0.899836<br>0.047522<br>0.020673
7	35539.6	281.4	0.004739834	H → L + 3<br>H → L + 4<br>H → L + 5	0.947827<br>0.023480<br>0.013067
8	36011.1	277.7	0.012585593	H - 4 → L<br>H - 3 → L<br>H → L + 8	0.910673<br>0.029860<br>0.010070
9	36915.5	270.9	0.022613247	H - 6 → L<br>H - 5 → L<br>H - 4 → L<br>H → L + 3<br>H → L + 4<br>H → L + 5<br>H → L + 7<br>H → L + 8	0.360435<br>0.013996<br>0.035708<br>0.023386<br>0.367391<br>0.100809<br>0.022878<br>0.051428
10	37479.6	266.8	0.001282627	H - 5 → L<br>H → L + 4	0.962862<br>0.021467

Table S6 – Vertical S_0–T_n (n = 1–10) transitions for **2b** in the ground state (TDA-PBE0-D3BJ/def2-TZVP(-f))

State	Energy, cm-1	Wavelength, nm	Transitions	Contributions
1	17594.5	568.4	H - 11 → L	0.014124 (c= -0.11884594)
			H → L	0.935783 (c= -0.96735878)
			H → L + 11	0.013670 (c= -0.11691752)
2	27349.0	365.6	H - 11 → L	0.010174 (c= -0.10086628)
			H - 6 → L	0.859386 (c= -0.92703051)
			H - 4 → L	0.070182 (c= -0.26491840)
			H - 3 → L	0.013551 (c= -0.11640871)
3	31527.6	317.2	H - 10 → L + 1	0.107595 (c= -0.32801634)
			H - 9 → L + 3	0.046255 (c= -0.21507045)
			H - 9 → L + 5	0.010413 (c= -0.10204213)
			H - 5 → L + 1	0.037735 (c= -0.19425617)
			H - 5 → L + 2	0.048696 (c= 0.22067107)
			H - 4 → L + 1	0.010132 (c= 0.10065818)
			H - 4 → L + 2	0.010970 (c= -0.10473612)
			H - 3 → L + 2	0.026889 (c= 0.16397937)
			H → L + 1	0.542336 (c= 0.73643452)
4	31702.3	315.4	H - 14 → L + 2	0.014598 (c= 0.12082307)
			H - 10 → L + 2	0.100599 (c= 0.31717317)
			H - 9 → L + 2	0.012345 (c= 0.11110817)
			H - 9 → L + 5	0.012501 (c= -0.11181007)
			H - 8 → L + 3	0.071204 (c= -0.26684070)
			H - 8 → L + 4	0.045671 (c= 0.21370822)
			H - 8 → L + 5	0.024985 (c= 0.15806766)
			H - 8 → L + 6	0.011319 (c= -0.10639256)
			H - 7 → L + 1	0.015903 (c= 0.12610695)
			H - 7 → L + 2	0.055871 (c= 0.23637134)
			H - 5 → L + 1	0.068874 (c= -0.26243887)
			H - 5 → L + 2	0.070674 (c= -0.26584612)
			H - 4 → L + 1	0.028387 (c= 0.16848507)
			H - 4 → L + 2	0.045274 (c= 0.21277717)
			H - 3 → L + 1	0.041061 (c= -0.20263391)
			H - 3 → L + 2	0.112287 (c= -0.33509290)
			H - 1 → L + 2	0.018613 (c= -0.13642815)
			H → L + 1	0.022644 (c= 0.15048027)
			H → L + 2	0.040489 (c= -0.20121949)
			H → L + 3	0.011114 (c= -0.10542513)
5	32385.8	308.8	H - 9 → L + 6	0.014713 (c= 0.12129552)
			H - 7 → L + 4	0.018323 (c= -0.13536343)
			H - 5 → L + 4	0.066675 (c= -0.25821439)
			H - 5 → L + 6	0.072595 (c= -0.26943385)
			H - 4 → L + 4	0.026340 (c= 0.16229647)
			H - 4 → L + 5	0.104959 (c= -0.32397322)
			H - 4 → L + 6	0.152378 (c= -0.39035681)
			H - 3 → L + 1	0.014251 (c= 0.11937591)
			H - 3 → L + 3	0.031606 (c= 0.17778059)

			H - 3 → L + 4<br>H - 3 → L + 5<br>H - 3 → L + 8<br>H - 1 → L + 4	0.196203 (c= 0.44294783)<br>0.083504 (c= -0.28897044)<br>0.010503 (c= -0.10248545)<br>0.011433 (c= -0.10692503)
6	32479.5	307.9	H - 19 → L<br>H - 18 → L<br>H - 14 → L<br>H - 11 → L<br>H - 10 → L<br>H - 10 → L + 1<br>H - 9 → L + 3<br>H - 8 → L<br>H - 6 → L<br>H - 5 → L + 1<br>H - 5 → L + 2<br>H - 3 → L<br>H → L + 1<br>H → L + 2<br>H → L + 8<br>H → L + 11	0.044525 (c= -0.21101036)<br>0.011330 (c= -0.10644128)<br>0.046687 (c= -0.21607266)<br>0.283669 (c= 0.53260608)<br>0.029495 (c= 0.17174184)<br>0.050092 (c= 0.22381327)<br>0.027678 (c= 0.16636752)<br>0.012451 (c= -0.11158281)<br>0.010210 (c= -0.10104290)<br>0.024996 (c= 0.15810222)<br>0.017158 (c= -0.13099039)<br>0.026317 (c= 0.16222439)<br>0.040579 (c= 0.20144211)<br>0.056659 (c= 0.23803089)<br>0.036128 (c= 0.19007345)<br>0.076510 (c= -0.27660385)
7	32907.1	303.9	H - 19 → L<br>H - 14 → L<br>H - 11 → L<br>H - 11 → L + 1<br>H - 10 → L<br>H - 10 → L + 1<br>H - 9 → L + 2<br>H - 9 → L + 3<br>H - 9 → L + 5<br>H - 5 → L + 1<br>H - 5 → L + 2<br>H - 4 → L + 1<br>H - 3 → L<br>H → L + 1<br>H → L + 8<br>H → L + 11	0.010497 (c= -0.10245411)<br>0.016764 (c= -0.12947510)<br>0.093416 (c= 0.30564021)<br>0.027674 (c= 0.16635471)<br>0.019225 (c= 0.13865532)<br>0.074792 (c= -0.27348093)<br>0.010954 (c= 0.10466217)<br>0.058215 (c= -0.24127884)<br>0.019601 (c= -0.14000471)<br>0.046206 (c= -0.21495543)<br>0.026314 (c= 0.16221459)<br>0.013278 (c= 0.11522905)<br>0.011540 (c= 0.10742283)<br>0.369421 (c= -0.60780009)<br>0.019943 (c= 0.14121963)<br>0.010925 (c= -0.10452221)
8	33549.6	298.1	H - 11 → L<br>H - 8 → L + 2<br>H - 1 → L<br>H → L + 2	0.018971 (c= -0.13773402)<br>0.016156 (c= 0.12710559)<br>0.013447 (c= 0.11596240)<br>0.849691 (c= 0.92178705)
9	34015.6	294.0	H - 3 → L<br>H - 2 → L<br>H - 1 → L<br>H → L + 8	0.013183 (c= -0.11481549)<br>0.028362 (c= -0.16841160)<br>0.888624 (c= 0.94266876)<br>0.020285 (c= 0.14242367)
10	34094.9	293.3	H - 4 → L<br>H - 3 → L	0.016353 (c= 0.12788041)<br>0.032695 (c= 0.18081668)

			H - 2 → L<br>H - 1 → L	0.849365 (c= 0.92160989)<br>0.038213 (c= 0.19548225)

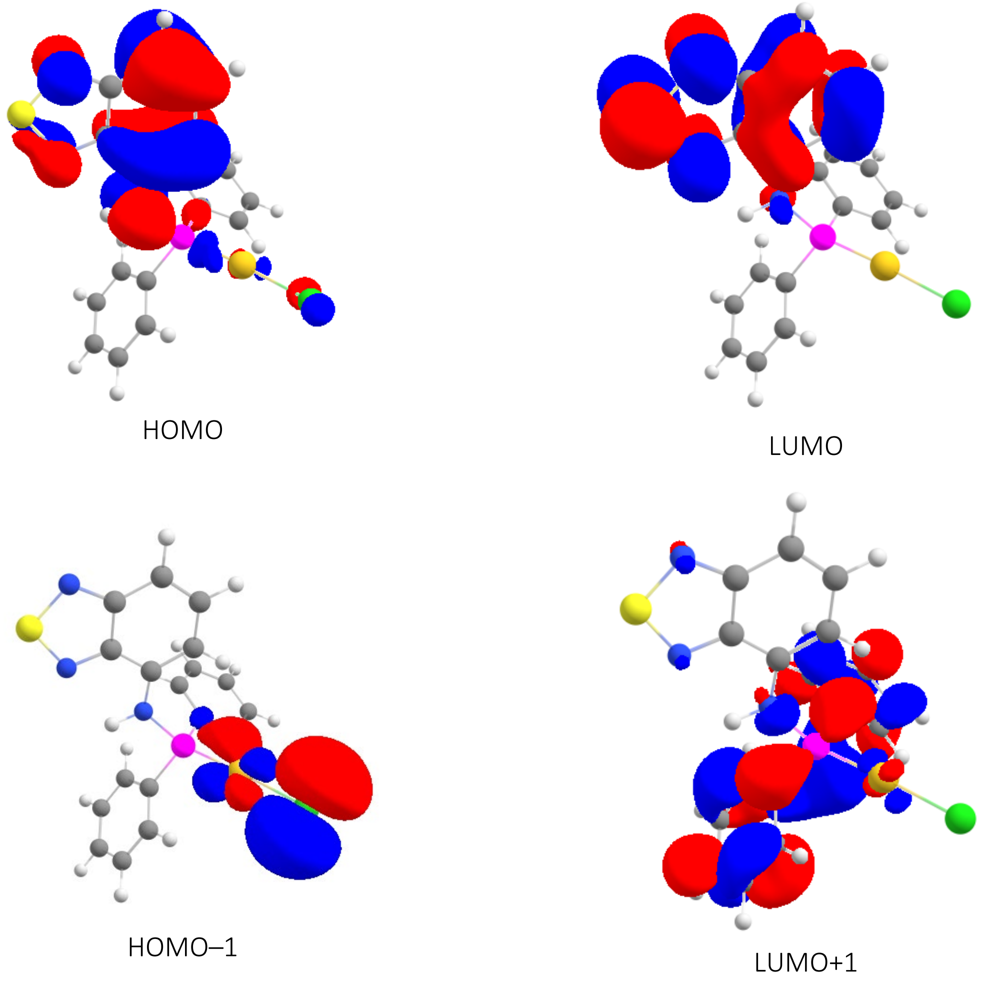

Figure S4. Frontier orbitals of S_0 equilibrium geometry of **1** (TD-PBE0-D3BJ/def2-TZVP(-f))

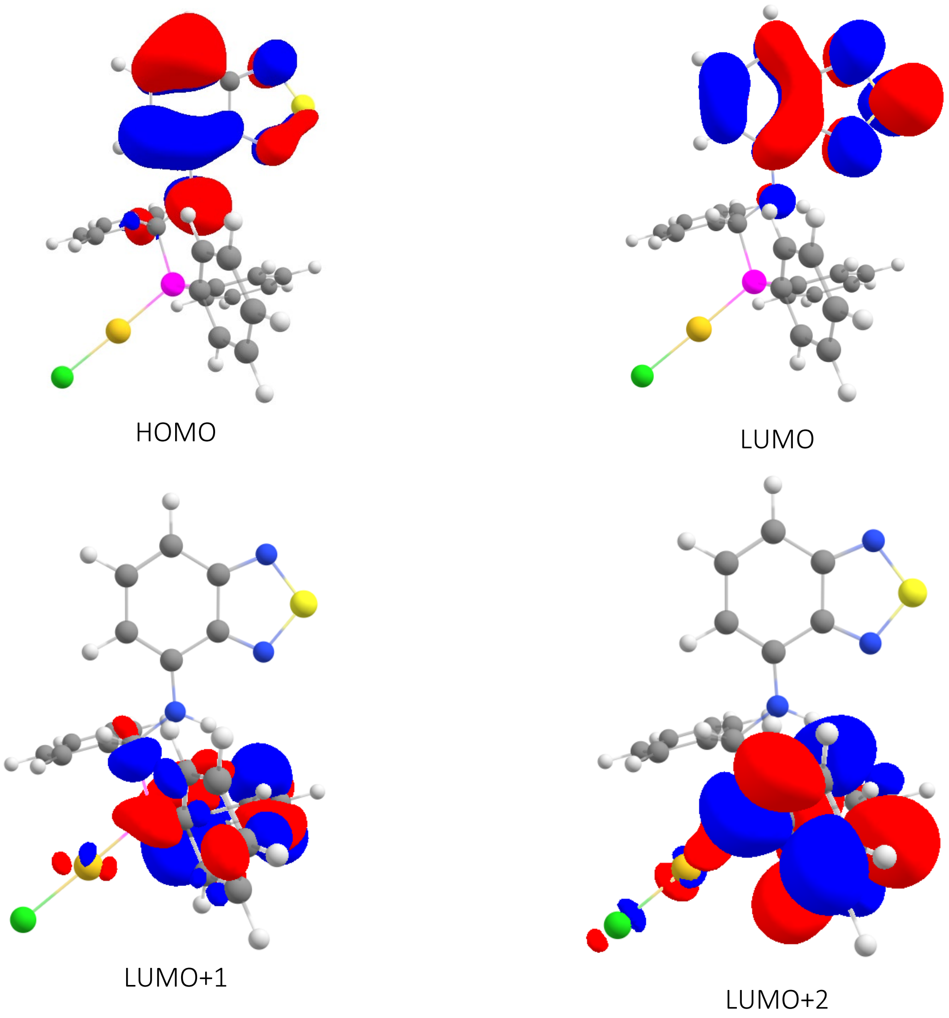

Figure S5. Frontier orbitals of S_0 equilibrium geometry of **2a** and **2b** on the example of the latter (TD-PBE0-D3BJ/def2-TZVP(-f))

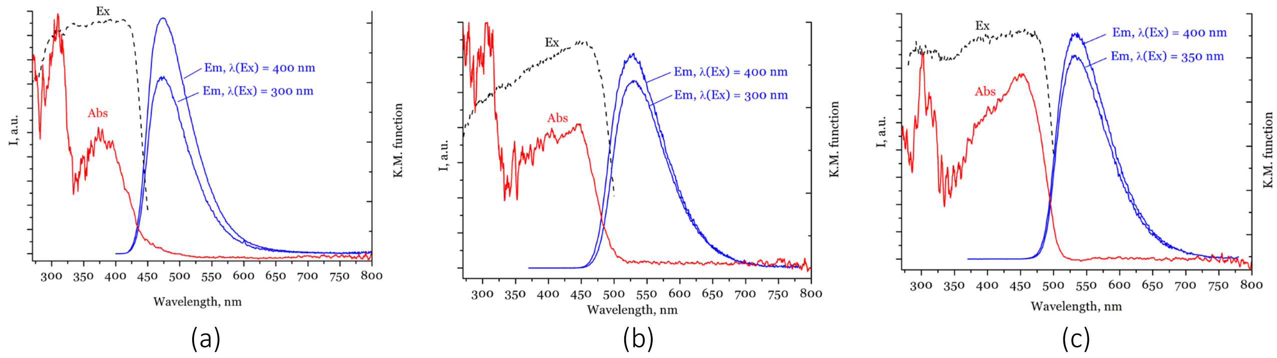

Figure S6. UV-Vis (red line), emission (blue lines; at different excitation wavelengths) and excitation (dashed black line) spectra of solids **1** (a), **2a** (b) and **2b** (c).

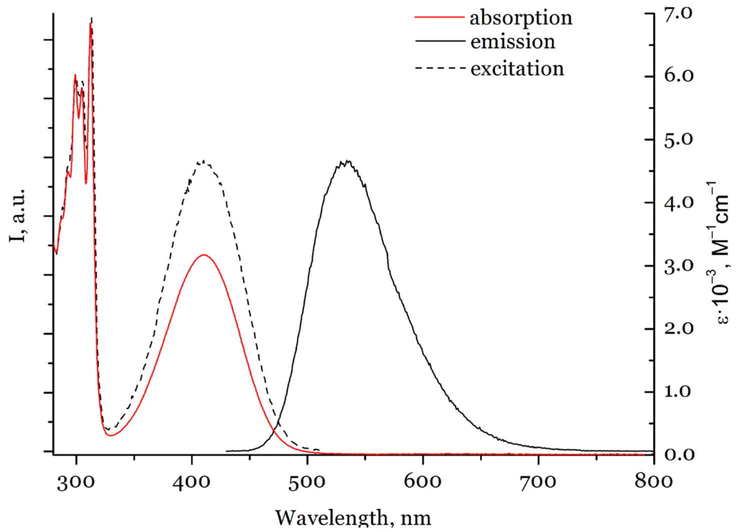

Figure S7. UV-Vis (red line), emission (solid black line; excitation wavelength 410 nm) and excitation (dashed black line) spectra of **2a** and **2b** in THF ($3{\cdot}10^{-5}$ M) on the example of the former.

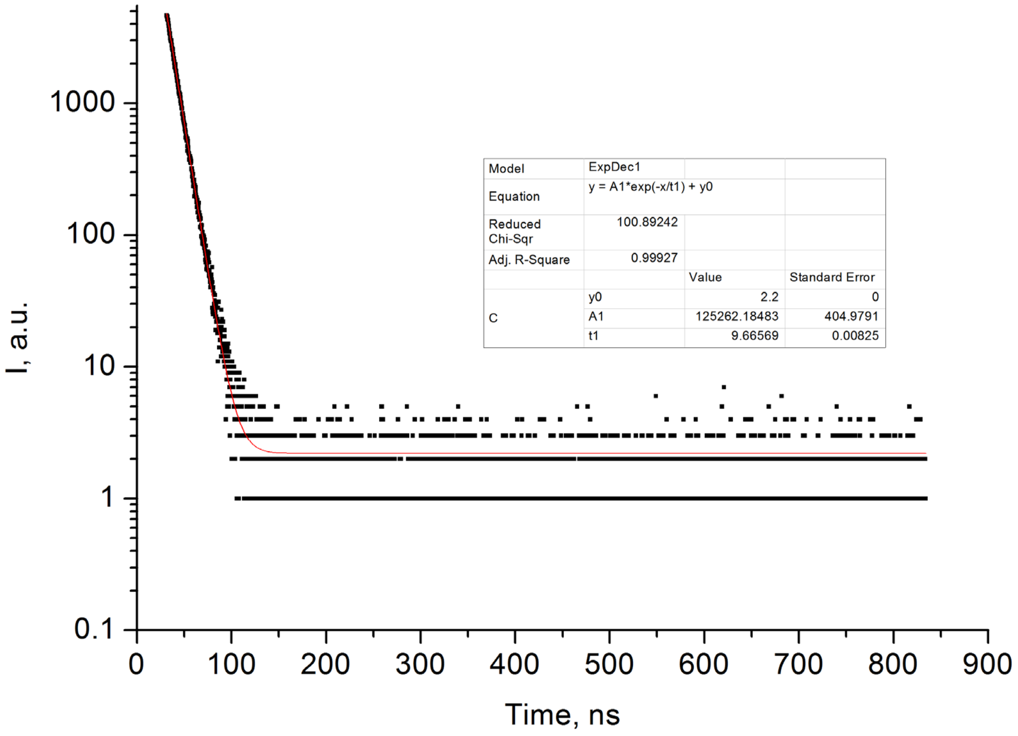

Figure S8. Emission decay kinetics (black dots) and single exponential fit (red line) for compound **1** (excitation wavelength 350 nm).

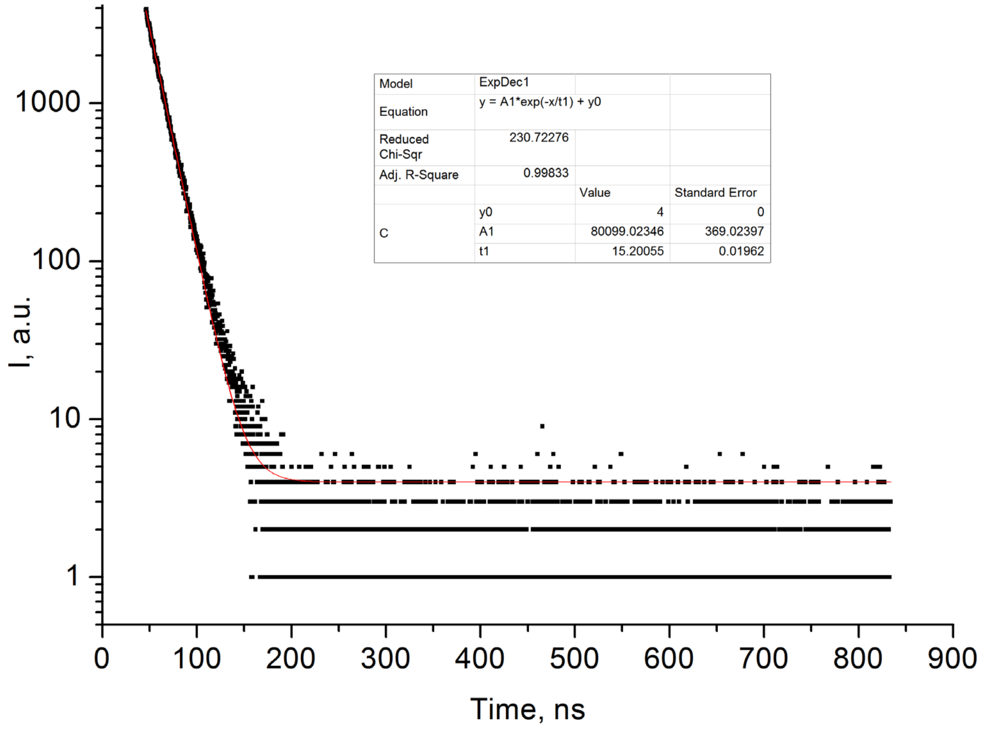

Figure S9. Emission decay kinetics (black dots) and single exponential fit (red line) for compound **2a** (excitation wavelength 350 nm).

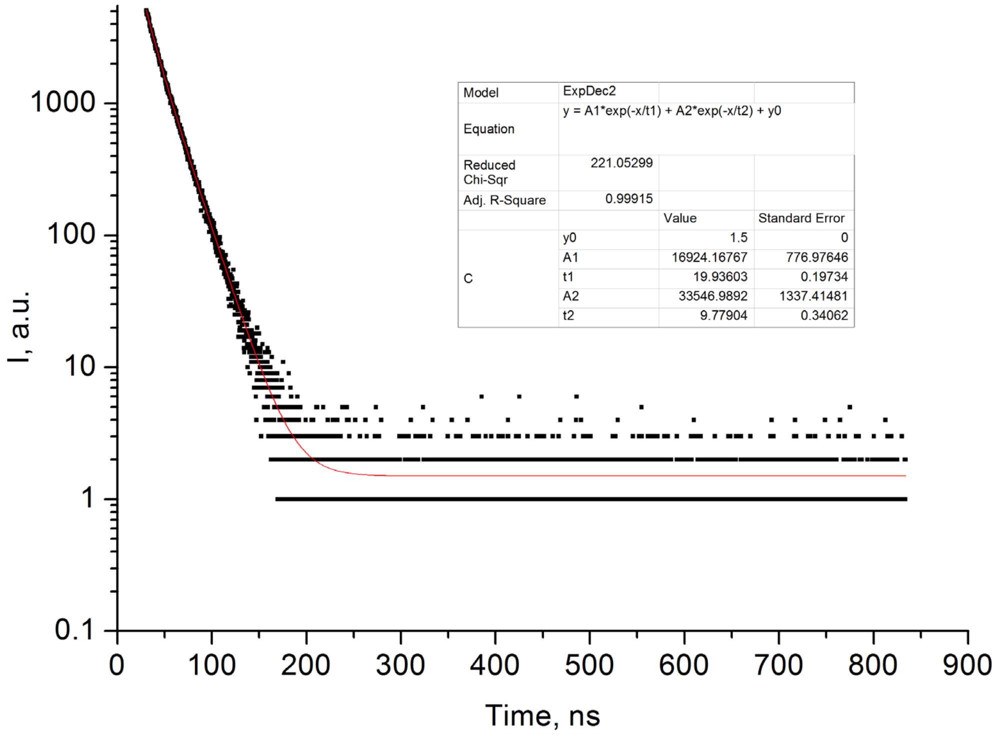

Figure S10. Emission decay kinetics (black dots) and double exponential fit (red line) for compound **2b** (excitation wavelength 350 nm).

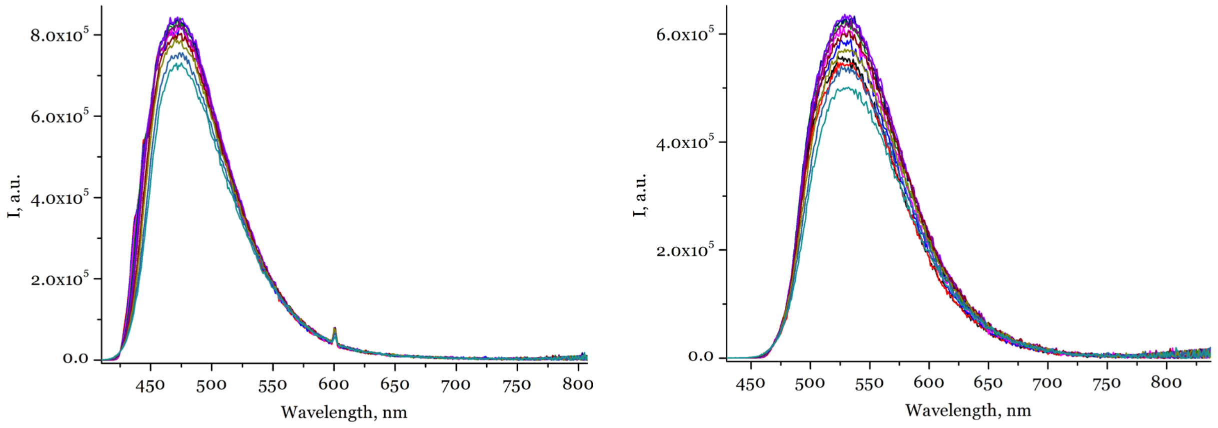

1 **2a**

Figure S11. Decreasing intensity of the emission bands for compounds **1** and **2a** in the temperature range 77–300 K at the excitation wavelength 300nm. A minor artefact at 600 nm for **1** is the second order Rayleigh scattering from the lamp. In the case of **2a**, it is hidden by the band of the compound.

Table S7. Values of selected spin-orbit coupling matrix elements for the complexes (cm^{-1}).

	**1**	**2a**	**2b**
$\langle T_1 \vert H_{SO} \vert S_0 \rangle$	16.8791	1.2554	2.0460
$\langle T_1 \vert H_{SO} \vert S_1 \rangle$	9.48840	0.3332	1.1093
$\langle T_1 \vert H_{SO} \vert S_2 \rangle$	23.0954	11.985	5.3687
$\langle T_2 \vert H_{SO} \vert S_0 \rangle$	16.1255	1.3537	0.8841
$\langle T_2 \vert H_{SO} \vert S_1 \rangle$	19.6617	1.0208	2.1309
$\langle T_2 \vert H_{SO} \vert S_2 \rangle$	5.31946	4.1497	1.2577

Table S8. Values of selected spin-orbit coupling matrix elements for complex **1** at excited states geometries (cm^{-1}).

	S_1	T_1	T_2
$\langle T_1 \vert H_{SO} \vert S_0 \rangle$	8.7789	9.7679	3.748506
$\langle T_1 \vert H_{SO} \vert S_1 \rangle$	4.0828	5.0321	11.27082
$\langle T_2 \vert H_{SO} \vert S_0 \rangle$	8.1901	13.6058	36.88823
$\langle T_2 \vert H_{SO} \vert S_1 \rangle$	11.3834	16.0571	36.93803

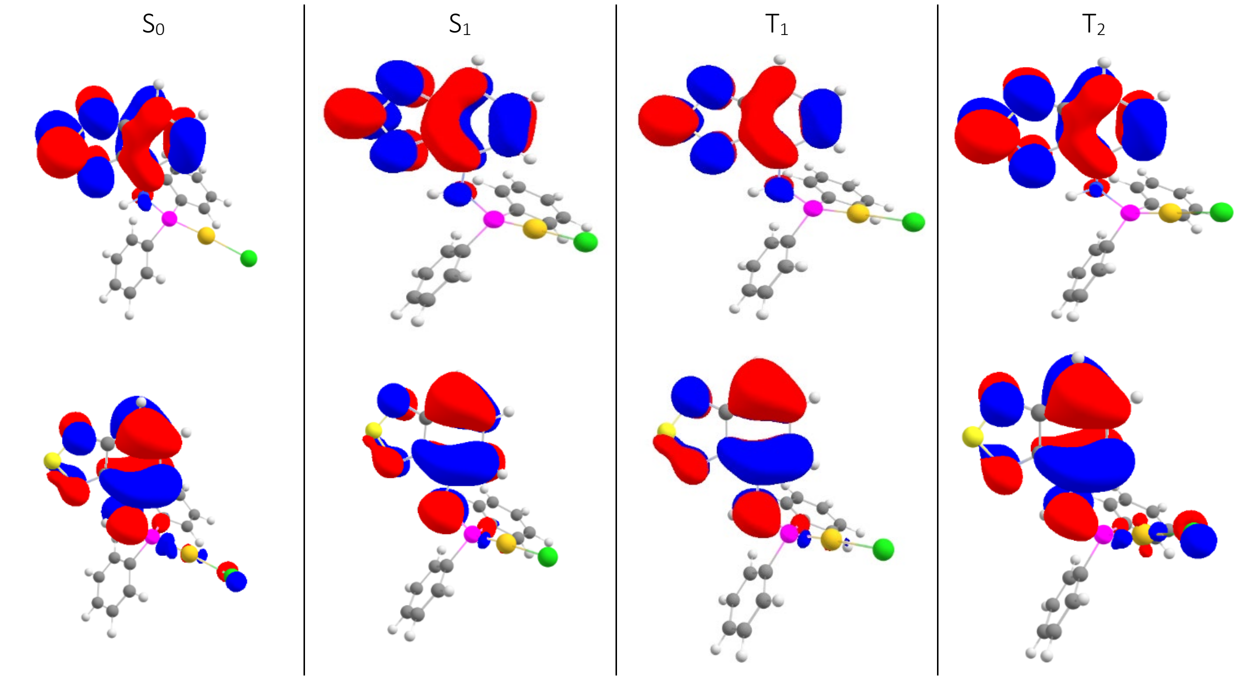

Figure S12. Comparison of LUMO (top) and HOMO (bottom) orbitals of **1** in S_0, S_1, T_1, and T_2 equilibrium geometries.

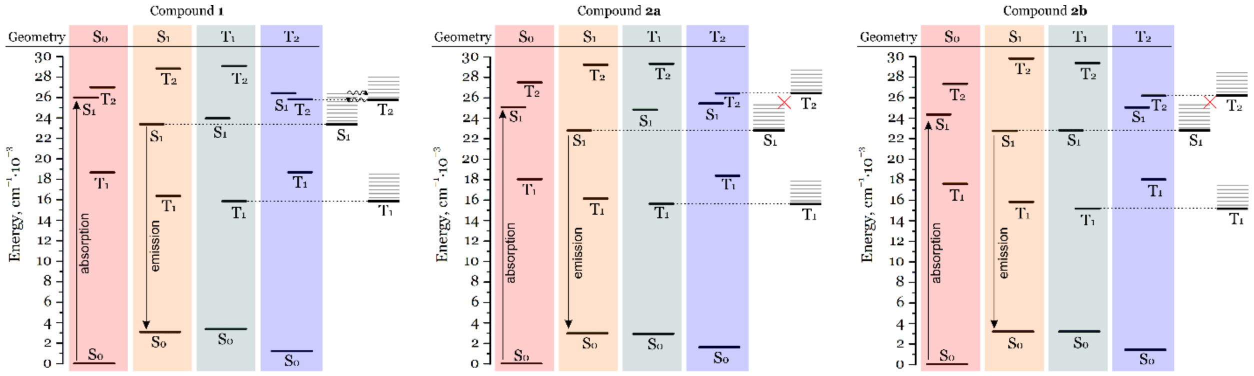

Figure S13. Representation of energy levels for different geometry states (S_o, S_1, T_1 and T_2) of complexes **1**–**2** in the energy range from 0 to 30·10^3 cm^{-1}.

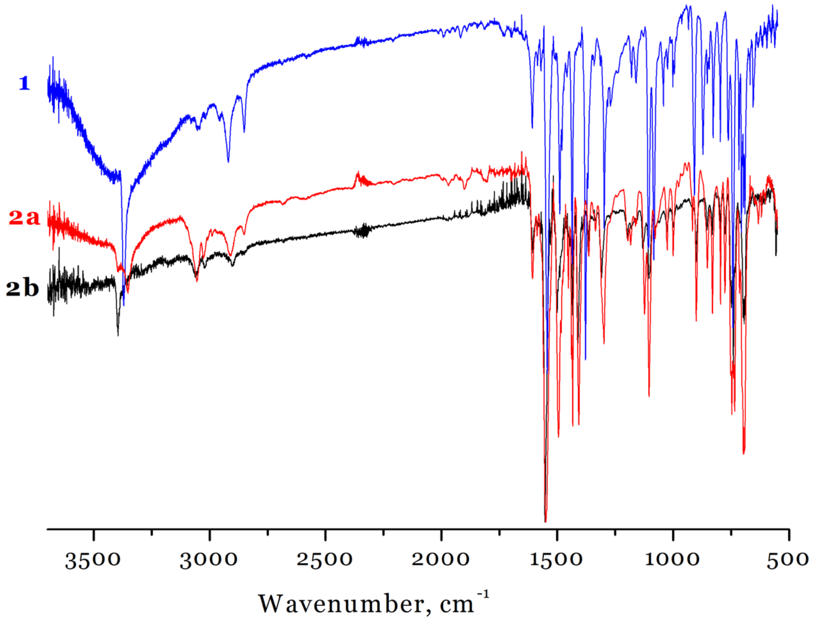

Figure S14. IR-spectra of compounds **1**–**2** recorded in KBr pellets

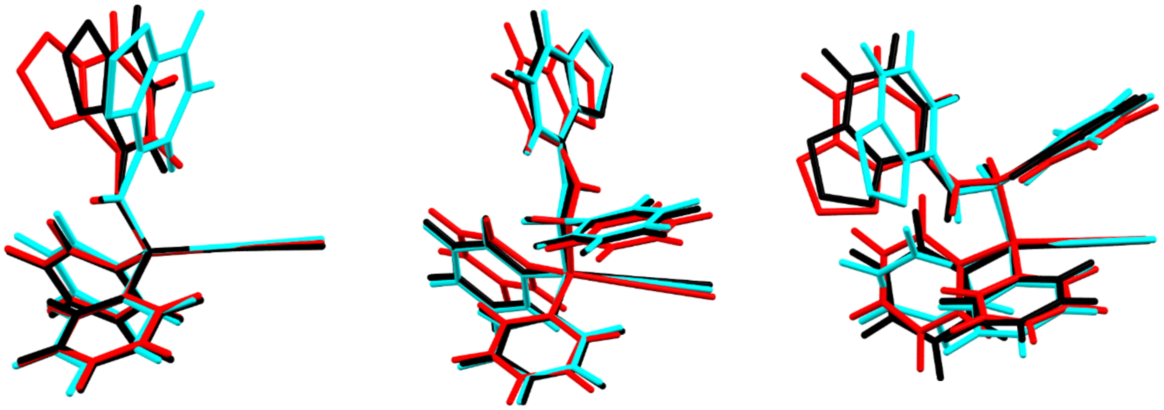

1 2a 2b

Figure S15. Comparison of equilibrium geometries of complexes **1**, **2a** and **2b** in different states. Black – S_0 state, red – S_1 state, blue – T_2 state.

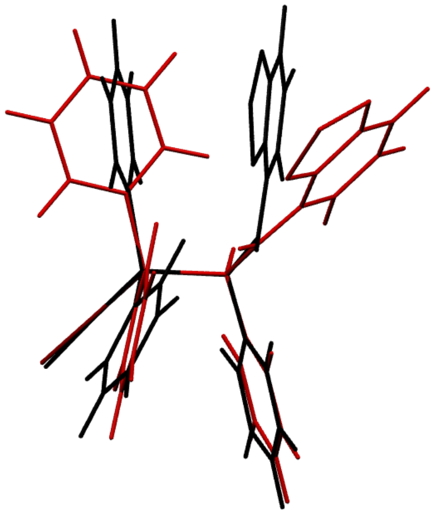

Figure S16. Overlaid geometries for S_1 equilibrium state of **2b** obtained by two different DFT methods. Black –PBE0-D3BJ/def2-TZVP(-f), red – BHandHLYP-D3BJ/def2-TZVP(-f)

Table S9. Crystal data and structure refinement for the compounds.

Identification code	**1**	**2a**	**2b**
Empirical formula	$C_{18}H_{14}AuClN_3PS$	$C_{25}H_{20}AuClN_3PS$	$C_{25}H_{20}AuClN_3PS$
Formula weight	567.77	657.89	657.89
Temperature/K	150(2)	150(2)	150(2)
Space group	$P2_12_12_1$	$C2/c$	$C2/c$
a/Å	10.1692(3)	34.9108(9)	22.1095(9)
b/Å	10.9529(3)	9.3689(2)	9.2709(5)
c/Å	16.5364(5)	14.9285(4)	23.4135(9)
β/°	90	94.9730(10)	103.4380(10)
Volume/Å^3	1841.86(9)	4864.4(2)	4667.8(4)
Z	4	8	8
ρ_{calc}g/cm^3	2.047	1.797	1.872
μ/mm^{-1}	8.338	6.328	6.595
F(000)	1080.0	2544.0	2544.0
Crystal size/mm^3	0.14 × 0.12 × 0.09	0.08 × 0.06 × 0.04	0.09 × 0.06 × 0.05
Radiation	MoKα (λ = 0.71073)	MoKα (λ = 0.71073)	MoKα (λ = 0.71073)
2Θ range for data collection/°	4.46 to 61.04	2.342 to 54.264	3.788 to 54.28
Index ranges	-14 ≤ h ≤ 14, -15 ≤ k ≤ 14, -23 ≤ l ≤ 23	-44 ≤ h ≤ 44, 0 ≤ k ≤ 12, 0 ≤ l ≤ 19	-28 ≤ h ≤ 27, -11 ≤ k ≤ 11, -30 ≤ l ≤ 30
Reflections collected	47766	5377	17773
Independent reflections	5642 [R_{int} = 0.0491, R_{sigma} = 0.0250]	5377 [R_{int} = 0.0639, R_{sigma} = 0.0223]	17773 [R_{int} = 0.0631, R_{sigma} = 0.0548]
Data/restraints/parameters	5642/1/229	5377/1/292	17773/1/293
Goodness-of-fit on F^2	1.074	1.055	1.043
Final R indexes [I>=2σ (I)]	R_1 = 0.0171, wR_2 = 0.0319	R_1 = 0.0276, wR_2 = 0.0615	R_1 = 0.0368, wR_2 = 0.0819
Final R indexes [all data]	R_1 = 0.0188, wR_2 = 0.0323	R_1 = 0.0359, wR_2 = 0.0643	R_1 = 0.0468, wR_2 = 0.0866
Largest diff. peak/hole / e Å^{-3}	0.59/-0.67	0.84/-0.82	0.74/-0.99
Flack parameter	-0.011(2)	–	–

Table S10. Selected bond lengths for structures **1**–**2**.

**1**			**2a**			**2b**		
Atom	Atom	Length/Å	Atom	Atom	Length/Å	Atom	Atom	Length/Å
Au1	Cl1	2.2833(9)	Au1	Cl1	2.2832(10)	Au1	Cl1	2.2856(13)
Au1	P1	2.2170(9)	Au1	P1	2.2301(10)	Au1	P1	2.2279(13)
S2	N1	1.623(4)	S2	N1	1.609(5)	S2	N1	1.614(5)
S2	N3	1.619(3)	S2	N3	1.617(4)	S2	N3	1.616(4)
P1	N4	1.672(3)	P1	C7	1.853(4)	P1	C7	1.865(5)
P1	C7	1.806(4)	P1	C14	1.810(4)	P1	C14	1.818(6)
P1	C13	1.806(3)	P1	C20	1.808(4)	P1	C20	1.814(5)

Table S11. Selected bond angles for structures **1**–**2**.

**1**				**2a**				**2b**			
Atom	Atom	Atom	Angle/°	Atom	Atom	Atom	Angle/°	Atom	Atom	Atom	Angle/°
P1	Au1	Cl1	176.60(4)	P1	Au1	Cl1	179.28(4)	P1	Au1	Cl1	176.71(5)
N3	S2	N1	100.95(16)	N1	S2	N3	101.5(2)	N1	S2	N3	100.6(2)
N4	P1	Au1	113.35(12)	C7	P1	Au1	110.92(13)	C7	P1	Au1	114.81(17)
N4	P1	C7	101.67(16)	C14	P1	Au1	114.21(14)	C14	P1	Au1	112.06(18)
N4	P1	C13	107.11(16)	C14	P1	C7	106.74(19)	C14	P1	C7	105.6(2)
C7	P1	Au1	113.04(12)	C20	P1	Au1	112.76(14)	C20	P1	Au1	112.27(17)
C7	P1	C13	105.86(16)	C20	P1	C7	104.63(18)	C20	P1	C7	106.4(2)
C13	P1	Au1	114.72(11)	C20	P1	C14	106.94(19)	C20	P1	C14	105.0(2)
C2	N1	S2	106.5(3)	C2	N1	S2	106.5(3)	C2	N1	S2	107.1(4)
C3	N3	S2	105.8(2)	C3	N3	S2	105.1(3)	C3	N3	S2	106.2(4)
C4	N4	P1	125.4(2)	N4	C7	P1	105.3(3)	N4	C7	P1	108.2(4)
				C8	C7	P1	110.7(3)	C8	C7	P1	111.7(3)
